# Time-Series Clustering of Single-Cell Trajectories in Collective Cell Migration

**DOI:** 10.3390/cancers14194587

**Published:** 2022-09-22

**Authors:** Zhuohan Xin, Masashi K. Kajita, Keiko Deguchi, Shin-ichiro Suye, Satoshi Fujita

**Affiliations:** 1 Department of Advanced Interdisciplinary Science and Technology, University of Fukui, Fukui 910-8507, Japan; 2 Department of Applied Chemistry and Biotechnology, University of Fukui, Fukui 910-8507, Japan; 3 Organization for Life Science Advancement Programs, University of Fukui, Fukui 910-8507, Japan; 4 Department of Frontier Fiber Technology and Science, University of Fukui, Fukui 910-8507, Japan

**Keywords:** collective cell migration, electrospinning, dimensionality reduction, clustering, migration pattern

## Abstract

**Simple Summary:**

In this study, we normalized trajectories containing both mesenchymal and epithelial cells to remove the effect of cell location on clustering, and performed a dimensionality reduction on the time series data before clustering. When the clustering results were superimposed on the trajectories prior to normalization, the results still showed similarities in location, indicating that this method can find cells with similar migration patterns. These data highlight the reliability of this method in identifying consistent migration patterns in collective cell migration.

**Abstract:**

Collective invasion drives multicellular cancer cells to spread to surrounding normal tissues. To fully comprehend metastasis, the methodology of analysis of individual cell migration in tissue should be well developed. Extracting and classifying cells with similar migratory characteristics in a colony would facilitate an understanding of complex cell migration patterns. Here, we used electrospun fibers as the extracellular matrix for the in vitro modeling of collective cell migration, clustering of mesenchymal and epithelial cells based on trajectories, and analysis of collective migration patterns based on trajectory similarity. We normalized the trajectories to eliminate the effect of cell location on clustering and used uniform manifold approximation and projection to perform dimensionality reduction on the time-series data before clustering. When the clustering results were superimposed on the trajectories before normalization, the results still exhibited positional similarity, thereby demonstrating that this method can identify cells with similar migration patterns. The same cluster contained both mesenchymal and epithelial cells, and this result was related to cell location and cell division. These data highlight the reliability of this method in identifying consistent migration patterns during collective cell migration. This provides new insights into the epithelial–mesenchymal interactions that affect migration patterns.

## 1. Introduction

Collective cell migration is a major mechanism of cell movement and is required for many physiological processes. This process is involved in biological processes, such as wound healing and tissue regeneration and several pathologies, including cancer metastasis. It relies on establishing polarized cell populations, which are asymmetrical in their front and rear. Polarization is caused by the presence of two types of cells, leader cells and follower cells, which differ in morphology and properties [1]. Leader cells with a large lamellipodial structure at the leading-edge guide follower cells through cell–cell interactions during collective migration [2]. This guidance has a prolonged effect on cell migration patterns. Although each cell migrates in different directions, once it becomes part of a population, mutual coordination between cells allows it to migrate quickly and to maintain adhesion with neighboring cells [3]. The complex mechanism of collective migration is remains unknown, but migration patterns resulting from cellular interactions must be thoroughly investigated.

Epithelial–mesenchymal transition (EMT) involves a series of biological changes in which epithelial cells lose their epithelial properties and acquire mesenchymal properties [4]. EMT-induced cells change in cell functions and phenotypes such as losing apical-basal polarity and gaining front–back polarity, changes in cell morphology, and acquisition of invasiveness due to the downregulation of epithelial genes and upregulation of mesenchymal genes [5,6,7]. These experimental observations support the idea that EMT-induced cells, along with the fibrous structure of the extracellular matrix (ECM), promote colony migration as leader cells. To analyze the interaction between the leader cells and other cells, studies that tracked the position of the leading edge of a single cell have elucidated the mechanism by which the deformation of intracellular microtubules affects migration [8,9]. How the positional relationship of cells in population changes has not been clarified. This study focused on the positional relationship of individual cells in a cell population to elucidate their migration patterns.

Cells migrate via actin polymerization at the leading edge of the colony to produce lamellipodia. For adhesion, they largely use a molecular mechanism involving integrin and related adhesion proteins [10,11,12]. In morphogenesis and cancer models, the loss of E-cadherin weakens cell connections, which causes cell detachment and migration during EMT [13,14]. E-cadherin is the main mediator of collective cell-cell interaction. The process of collective cell migration can be described to varying degrees using molecular mechanisms and mechanical models [15,16,17]. However, how these interactions integrate multiple factors that influence migration patterns requires further investigation [18]. We used a simpler approach based on the clustering of single-cell trajectories to explore the migration patterns between cells.

The study of cell migration patterns usually requires recording cell trajectories within observation windows comprising multiple sets of timeseries data. Timeseries data are characterized by high dimensionality and large data volumes [19]. Clustering such complex objects can reveal interesting patterns. As an unsupervised learning technique, k-means clustering is a commonly used clustering method. The main idea behind this clustering method is to minimize the total distance (usually the Euclidean distance) between all the objects in a cluster and their cluster centers. Cluster centers are defined as the average vectors of the objects in the cluster; however, time series clustering poses additional challenges. For cell trajectories, this increases the difficulty and computational effort of clustering based on the time dimension and poor clustering performance owing to the different locations of cell trajectories at each time record point, and similar problems have been observed in the phylogenetic analysis [20,21].

One solution is dimensionality reduction of time series data and subsequent clustering. Commonly used dimensionality reduction methods to maintain the two-by-two distance structure of the dataset include principal component analysis (PCA) [22], multi-dimensional scaling (MDS) techniques [23], and streamwise learning techniques such as t-distributed stochastic neighbor embedding (t-SNE), which can maintain the local structure of the data [24] and uniform manifold approximation and projection (UMAP) [25]. In particular, UMAP is efficient for processing large amounts of data in response to the global structure, providing higher-quality visualization. Clustering is often used in biological disciplines for the identification of functionally relevant genes and functional clustering of gene expression data [26,27]; however, to our knowledge, this is the first report to analyze the range of influence of leader cells on collective migration by classifying the movement pattern with clustering analysis of individual cell tracking.

**Scheme 1:**
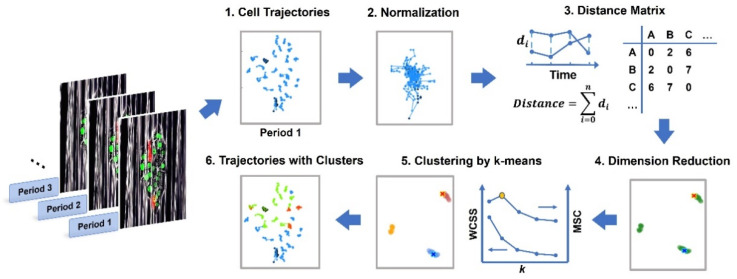
Schematic of time-series clustering of cell trajectories in collective cell migration. After collecting cell migration movies by time-lapse microscopy, cell trajectories were generated manually by period using ImageJ. Second, the trajectories of each period were normalized to convert their starting points to the origin (0, 0). Third, the distance matrix is calculated from the Euclidean distance of each pair of trajectories. Fourth, the UMAP dimensionality reduction algorithm is used to visualize the data in two dimensions. Fifth, the data are clustered by k-means, where the number of clusters is determined by the mean silhouette coefficient. Finally, the clusters are combined with the original trajectories to analyze similar migration patterns in collective cell migration.

In this study, we attempted to identify similar migration patterns among cells (Scheme 1). As an approach to quantitatively analyze cell–cell interactions, co-culture of EMT-induced mesenchymal cells by the addition of transforming growth factor β1 (referred as TGF (+)) and EMT non-induced cells (TGF (−)) was performed to imitate colony migration on aligned electrospun fibers mimicking the ECM fibrous architecture. Tracking of cell migration by time-lapse observation followed by dimensionality reduction and clustering was performed to find similar migration patterns, where they are not only related to location but also influenced by cell division.

## 2. Materials and Methods

### 2.1. Electrospinning

To prepare the cell culture scaffold, 20 wt% polystyrene (PS) solutions dissolved in tetrahydrofuran (THF) were electrospun on a cover slip (18 × 24 mm) using a commercial electrospinning setup (NANON, MECC, Fukuoka, Japan) under the following conditions: flow rate, 0.1 mL/h; linear velocity of collector rotation, 15.7 m/s; electric field, 2.5 kV/cm; spinning time, 60 min. The electrospun fibers were treated with O_2_ plasma (40 kHz/100 W, 25 Pa, 30 s) and coated with 10 μg/mL fibronectin (37 °C, 2 h).

### 2.2. Cell Culture and Time-Lapse Observation

We used the murine mammary gland cell line NMuMG (ATCC CRL-1636) and fluorescently labeled NMuMG cells (NMuMG-DsRed). The gene induction of pDsRed2-C1 was conducted according to the guidelines of the ethics committee of the institution. Cells were maintained in DMEM supplemented with 10% FBS at 37 °C, 5% CO_2_, and 95% humidity. EMT was induced by adding 10 ng/mL TGF-β1 for 3 days [28]. To prepare cell aggregates, 500 μL of the cell suspension of non-labeled NMuMG cells (designated as TGF (−) cells) and EMT-induced NMuMG-DsRed cells (designated as TGF (+) cells) at a ratio of 1:4 at 1 × 10^5^ cells/mL were seeded onto a 24-well grided-plate (Elplasia #4445; Kuraray, Tokyo, Japan) and incubated for 3 h. Cell aggregates formed in the grids were harvested by gentle pipetting. They were suspended in 500 μL of medium containing 25 μL of CellLightTM Histone 2B-GFP and BacMam 2.0, (Thermo Fisher Scientific K.K., Tokyo, Japan), inoculated on fibronectin-coated fibers, cultured for 24 h, and then used for the migration assay. Time-lapse microscopy was performed for 24 h at intervals of 15 min using Biostation (Nikon, Tokyo, Japan) and analyzed using ImageJ (ver. 1.53 e). The captured fluorescence images were binarized and the cells were tracked using a plug-in (TrackMate). The location of the center of the cell nucleus was determined by manual tracking which started from the last time slice to the first time slice backward. The cell was identified on the basis of the locations of the cell nucleus between two adjacent slices and assigned ID numbers to all cells. The slice when cell division occurred was determined by comparing the distance between neighboring cell marker points.

### 2.3. Cell Trajectory Data and Its Normalization

Let

(1) $$V_{i}^{\left(p\right)}≔\langle v_{i,1}^{\left(p\right)},\ldots ,v_{i,n}^{\left(p\right)}\rangle$$

be the ith cell’s trajectory of the pth time period, where $v_{i,l}^{\left(p\right)}\in$ ${\mathbb{R}}^{2}$ is the cell position at lth time slice of the pth time period and n is the number of time slices in a single time period.

The cells were divided several times during the observation. After manually marking the positions of the cell nuclei and finding mother cells with daughter cells, the trajectories of two daughter cells were unified with the mother cell prior to division. To remove the effect of the initial cell positions from the cell trajectory clustering, we first normalized the ith cell’s trajectory of pth time period $V_{i}^{\left(p\right)}$ such that its initial position $v_{i,l}^{\left(p\right)}$ was located at the origin (0,0). The normalized trajectory is given by

(2) $$Z_{i}^{\left(p\right)}≔\langle z_{i,1}^{\left(p\right)},\ldots ,z_{i,n}^{\left(p\right)}\rangle ,$$

where $z_{i,l}^{\left(p\right)}≔v_{i,l}^{\left(p\right)}-v_{i,1}^{\left(p\right)}={\left(z_{i,l,1}^{\left(p\right)},z_{i,l,2}^{\left(p\right)}\right)}^{⊤}$.

### 2.4. Distance Matrix of Cell Trajectories

To evaluate the similarity of cell trajectories, we defined the pairwise distance between two trajectories as follows: the distance between two trajectories at the pth time period, $Z_{i}^{\left(p\right)}$ and $Z_{j}^{\left(p\right)}$, is defined by

(3) $$D\left(Z_{i}^{\left(p\right)},Z_{j}^{\left(p\right)}\right)≔\overset{n}{\underset{l=1}{\sum }}\delta \left(z_{i,l}^{\left(p\right)},z_{j,l}^{\left(p\right)}\right),$$

where $\delta \left(z_{i,l}^{\left(p\right)},z_{j,l}^{\left(p\right)}\right)$ is the distance between two points, $z_{i,l}^{\left(p\right)}$ and $z_{j,l}^{\left(p\right)}$, which are respectively ith and jth cell positions at lth time slice of pth time period.

In this study, we employed the Euclidean distance to define the distance function $\delta \left(z_{i,l}^{\left(p\right)},z_{j,l}^{\left(p\right)}\right)$:

(4) $$\delta \left(z_{i,l}^{\left(p\right)},z_{j,l}^{\left(p\right)}\right)≔\sqrt{{\left(z_{i,l,1}^{\left(p\right)}-z_{j,l,1}^{\left(p\right)}\right)}^{2}+{\left(z_{i,l,2}^{\left(p\right)}-z_{j,l,2}^{\left(p\right)}\right)}^{2}.}$$

Based on the pairwise distance between the two trajectories $D\left(Z_{i},Z_{j}\right)$, the distance matrix is defined as follows:

(5) $$D≔\left[\begin{array}{ccc} D_{1,1} & \cdots & D_{1,N}\\ \vdots & \ddots & \vdots \\ D_{N,1} & \cdots & D_{N,N} \end{array}\right],$$

where $D_{i,j}≔D\left(Z_{i},Z_{j}\right)$ and N is the total number of cells and their trajectories.

### 2.5. Two-Dimensional Representation of Cell Trajectories Based on a Distance Matrix

To visualize the similarity of the cell trajectories, we embedded each cell trajectory $Z_{i}^{\left(p\right)}$ into a two-dimensional space based on the distance matrix D. We employed t-SNE [24,29,30,31] and UMAP [25] as the embedding methods using Python (scikit-learn 1.1.2; umap-learn 0.5.3). By applying these two methods, we obtain a set of cell trajectories in pth time period represented in a two-dimensional space:

(6) $$D^{\left(p\right)}≔\left\{x_{1}^{\left(p\right)},\ldots ,x_{N}^{\left(p\right)}\right\},$$

where $x_{i}^{\left(p\right)}\in {\mathbb{R}}^{2}$ is the ith cell trajectory of the pth time period in two-dimensional space.

### 2.6. Clustering of Cell Trajectories

Next, we classified cell trajectories based on their similarities. Here, we employed the k-means clustering method [32] for classification. Using k-means clustering, we partitioned the N samples of $D^{\left(p\right)}$ into $K_{p}\left(\leq N\right)$ sets of the pth time period $M_{p}=\left\{M_{1}^{\left(p\right)},M_{2}^{\left(p\right)},\ldots ,M_{K_{p}}^{\left(p\right)}\right\}$ based on the similarities among the samples.

Let

(7) $${ℳ}_{p}=\left\{\mu_{1}^{\left(p\right)},\ldots ,\mu_{K_{p}}^{\left(p\right)}\right\}$$

be a set of the mean of points in each cluster at the pth time period, where $\mu_{k}^{\left(p\right)}$ is the mean of points in the kth cluster at the pth time period. We also introduce a variable that denotes whether a set $M_{k}^{\left(p\right)}$ contains $x_{i}^{\left(p\right)}$ or not:

(8) $$q_{i,k}^{\left(p\right)}=\{\begin{array}{ll} 1 & \left(x_{i}^{\left(p\right)}\in M_{k}^{\left(p\right)}\right),\\ 0 & \left(x_{i}^{\left(p\right)}\notin M_{k}^{\left(p\right)}\right). \end{array}$$

Using the variables, the objective function of k-means clustering, the within-cluster sum of squares (WCSS), is defined as

(9) $$J\left(q_{i,k}^{\left(p\right)},\mu_{k}^{\left(p\right)}\right)=\overset{N}{\underset{i=1}{\sum }}\overset{K_{p}}{\underset{k=1}{\sum }}q_{i,k}^{\left(p\right)}∥x_{i}^{\left(p\right)}-\mu_{k}^{\left(p\right)}{∥}^{2}.$$

The optimal solution is a parameter that minimizes the objective function $J\left(q_{i,k}^{\left(p\right)},\mu_{k}^{\left(p\right)}\right)$. We numerically solved the optimization problem using the k-means algorithm. Notably, the solution of the k-means algorithm has an initial value dependency. Therefore, we tested several initial values and employed a clustering result that minimized the objective function.

The number of clusters at the pth time period $K_{p}$ is a hyperparameter of k-means clustering. Therefore, we optimized $K_{p}$ based on the silhouette analysis [33]. Silhouette analysis evaluates clustering results based on the degree of aggregation within clusters and the degree of separation between clusters.

The silhouette coefficients of the samples were calculated at different $K_{p}$ values. This coefficient measures how similar an object is to its cluster compared with other clusters. After the samples were clustered into $K_{p}$ clusters, the average distance of $x_{i}^{\left(p\right)}$ from the other samples in the cluster was calculated for the sample $x_{i}^{\left(p\right)}$ in cluster $M_{I}^{\left(p\right)}$. Within-cluster dissimilarity, which measures how well $x_{i}^{\left(p\right)}$ is assigned to its cluster, is defined as follows:

(10) $$a\left(x_{i}^{\left(p\right)}\right)=\frac{1}{\left|M_{I}^{\left(p\right)}\right|-1}\underset{j\in M_{I}^{\left(p\right)},j\neq i}{\sum }\delta \left(x_{i}^{\left(p\right)},x_{j}^{\left(p\right)}\right),$$

where $\left|M_{I}^{\left(p\right)}\right|$ is the number of samples belonging to cluster *I*. The definition of the distance function is the same as that of the distance function in Equation (4).

The between-cluster dissimilarity, the average distance between $x_{i}^{\left(p\right)}$ and all samples in the other cluster $M_{J}^{\left(p\right)}$, are defined as follows:

(11) $$b\left(x_{i}^{\left(p\right)}\right)=\underset{J\neq I}{\min}\frac{1}{\left|M_{J}^{\left(p\right)}\right|}\underset{j\in M_{J}^{\left(p\right)}}{\sum }\delta \left(x_{i}^{\left(p\right)},x_{j}^{\left(p\right)}\right).$$

The silhouette coefficient of sample $x_{i}^{\left(p\right)}$ is defined as follows:

(12) $$s\left(x_{i}^{\left(p\right)}\right)=\{\begin{array}{ll} 1-\frac{a\left(x_{i}^{\left(p\right)}\right)}{b\left(x_{i}^{\left(p\right)}\right)}, & \;\mathrm{if}\;a\left(x_{i}^{\left(p\right)}\right)<b\left(x_{i}^{\left(p\right)}\right)\\ 0, & \;\mathrm{if}\;a\left(x_{i}^{\left(p\right)}\right)=b\left(x_{i}^{\left(p\right)}\right)\\ \frac{b\left(x_{i}^{\left(p\right)}\right)}{a\left(x_{i}^{\left(p\right)}\right)}-1, & \;\mathrm{if}\;a\left(x_{i}^{\left(p\right)}\right)>b\left(x_{i}^{\left(p\right)}\right) \end{array}$$

The silhouette coefficient $s\left(x_{i}^{\left(p\right)}\right)$ is a measure of how well the data are clustered over the entire dataset. A larger value indicates a more reasonable clustering of $x_{i}^{\left(p\right)}$.

Based on the silhouette coefficients, we obtained the optimal number of clusters $K_{p}$:

(13) $${\hat{K}}_{p}=\underset{K_{p}}{\arg\max}\bar{s}\left(K_{p}\right),$$

where $\bar{s}\left(K_{p}\right)$ is the mean silhouette coefficient (MSC), defined as

(14) $$\bar{s}\left(K_{p}\right)≔\frac{1}{N}\overset{N}{\underset{i=1}{\sum }}s(x_{i}^{\left(p\right)}).$$

The above equation represents the mean $s\left(x_{i}^{\left(p\right)}\right)$ over all cell trajectories for a specific number of clusters $K_{p}$. Cell–cell interactions can change during migration, and cell division also perturbs these interactions. Thus, the number of clusters $K_{p}$ may vary over time. Therefore, this process is repeated for each period p and is used to determine the appropriate number of clusters ${\hat{K}}_{p}$.

### 2.7. Robustness of Our Method

Our method depends on the accuracy of the cell tracking data, particularly the accuracy of the estimated positions of the cell nucleus center. In this study, the positions of the cell nucleus centers were detected manually from bright field images, which might have been affected by the observation noise. Thus, we evaluated the robustness of our clustering method to the intensity of observation noise in the estimated cell nucleus positions.

As shown in Equation (1) $V_{i}^{\left(p\right)}=\langle v_{i,1}^{\left(p\right)},\ldots ,v_{i,n}^{\left(p\right)}\rangle$ denotes the cell trajectories, where $v_{i,l}^{\left(p\right)}\in {\mathbb{R}}^{2}$ is the cell position at the *l*th time slice of the pth time period and n is the number of time slices in a single time period.

We assume that the observation noise of the cell nucleus center position is modeled as two-dimensional Gaussian noise as follows:

(15) $$\left\{\xi_{l}\right\}\overset{\;i.i.d.\;}{∼}N_{2}\left(0,\Sigma \right).$$

Here, $\xi_{l}\in {\mathbb{R}}^{2}$ and $N_{2}\left(0,\Sigma \right)$ denote a two-dimensional Gaussian distribution, where the mean 0,Σ denotes the variance–covariance matrix, which is defined as

(16) $$\Sigma =\left[\begin{array}{ll} \sigma & 0\\ 0 & \sigma \end{array}\right].$$

The true nucleus center $v_{i,l}^{\left(p\right)}$ is unknown, but we assume that $v_{i,l}^{\left(p\right)}$ is the true center. We then generated the test dataset by adding Gaussian noise to the original single-cell trajectory dataset, as follows:

(17) $$V_{i}^{\prime (p)}=\langle v_{i,1}^{\left(p\right)}+\xi_{1},\ldots ,v_{i,n}^{\left(p\right)}+\xi_{n}\rangle .$$

Next, we evaluated the consistency of the clustering results obtained from the datasets, original dataset, and generated test datasets. Let $n\left(M_{k}^{\left(p\right)}\right)$ be the number of elements in the kth cluster at the pth time period, and $n^{\left(p\right)}$ denote a vector whose element is $n\left(M_{k}^{\left(p\right)}\right)$ for $1\leq k\leq K_{p}$, which is defined as

(18) $$n^{\left(p\right)}≔{\left(n\left(M_{1}^{\left(p\right)}\right),n\left(M_{2}^{\left(p\right)}\right),\ldots ,n\left(M_{K_{p}}^{\left(p\right)}\right)\right)}^{⊤},$$

where $n\left(M_{k}^{\left(p\right)}\right)$ for $1\leq k\leq K_{p}$ is sorted in descending order; thus, it satisfies $n\left(M_{k^{\prime }}^{\left(p\right)}\right)\geq n\left(M_{k^{\prime }+1}^{\left(p\right)}\right)$.

Let $n_{u}^{\left(p\right)}$ be $n^{\left(p\right)}$ obtained from data $D_{u}^{\left(p\right)}$, where $D_{u}^{\left(p\right)}$ denotes a set of cell trajectories in the pth time period represented in two-dimensional space (see Equation (6)), u≥1. u=0 denotes the index of the original dataset, and u≥1 denotes that of the test dataset generated by adding Gaussian noise to the original dataset. The consistency of the clustering results obtained from the original and test datasets is defined by the Euclidean distance between the clustering results obtained from the two datasets. Before calculating the consistency, we normalized $n_{u}^{\left(p\right)}$ by the total number of cells in the pth time slice $N^{\left(p\right)}$. The normalized clustering result is defined as follows:

(19) $$n_{u}^{\prime \left(p\right)}≔{\left(\frac{n_{u}\left(M_{1}^{\left(p\right)}\right)}{N^{\left(p\right)}},\ldots ,\frac{n_{u}\left(M_{K_{p}}^{\left(p\right)}\right)}{N^{\left(p\right)}}\right)}^{⊤}.$$

Then, the consistency between the original dataset and the test dataset is defined by the Euclidean distance between $n_{0}^{\prime \left(p\right)}$ and $n_{u}^{\prime \left(p\right)}\left(u\geq 1\right)$, which is

(20) $$\delta \left(n_{0}^{\prime (p)},n_{u\geq 1}^{\prime (p)}\right)=\sqrt{\overset{K_{p}}{\underset{k=1}{\sum }}{\left(\frac{n_{0}\left(M_{k}^{\left(p\right)}\right)}{N^{\left(p\right)}}-\frac{n_{u\geq 1}\left(M_{k}^{\left(p\right)}\right)}{N^{\left(p\right)}}\right)}^{2}}.$$

We generated several test datasets (u≥1) using Equation (17) and obtained a set of test dataset clustering results.

(21) $${ℛ}^{\left(p\right)}≔\left\{n_{1}^{\left(p\right)},n_{2}^{\left(p\right)},\ldots ,n_{h}^{\left(p\right)}\right\},$$

where *h* denotes the number of the test datasets. Finally, we defined the difference score as the mean value of $\delta \left(n_{0}^{\prime \left(p\right)},n_{u\geq 1}^{\prime \left(p\right)}\right)$ for u≥1 as follows. A lower difference score indicates a better robustness.

(22) $${\bar{\delta }}_{{ℛ}^{\left(p\right)}}≔\frac{1}{h}\overset{h}{\underset{u=1}{\sum }}\delta (n_{0}^{\prime (p)},n_{u}^{\prime (p)}).$$

## 3. Results and Discussion

### 3.1. Cell Tracking

**Figure 1:**
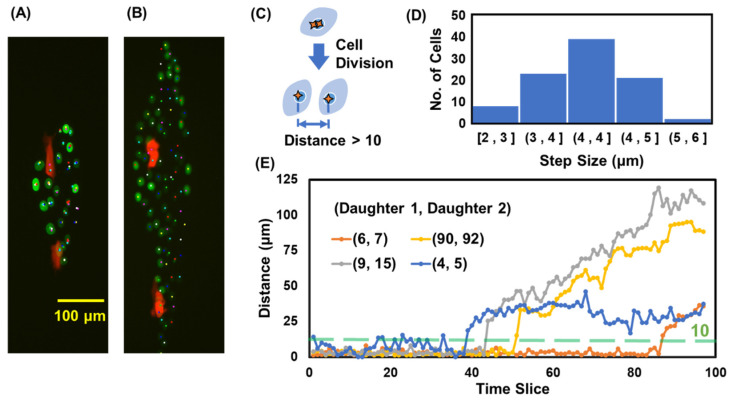
Daughter cell pairs were identified by calculating the “distance jump” between the markers. (**A**,**B**) Manual markers of cell position at time slice 1 and 96. (**C**) Schematic of calculation of distance between the marker of mother and daughter cell. (**D**) The distribution of step size (distance between two adjacent slices) of each cell before division. (**E**) Euclidean distance between two daughter cells.

The interaction between mesenchymal and epithelial cells is an important driving force for collective cell migration. Interactions promote the transmission of signals between cells, which guides the migration of follower cells. Thus, multiple cells follow the leader’s migration. Here, the influence of leader cells was investigated by observing the cell population in a model in which TGF (+) cells (red labeled) and TGF (−) cells coexist (Figure 1A, Movie S1). Figure 1A,B indicate that the shape of the colony changed gradually from a circle to an ellipse, suggesting that the cell migration was not random but anisotropic due to the aligned electrospun fibers. The migration of each cell was one-directional toward the outside of the colony, which might be due to the local gradient of the soluble factors secreted by cells because the cell culture medium had no intentional mechanism to cause a chemotactic gradient; it is beyond the scope of this study but warrants further investigation.

At this time, TGF (+) cells were labeled with the red fluorescent protein DsRed to distinguish them from TGF (−). A population of 10–20 cells was observed because its size was the same as that of a colony in vivo. In the first time slice, there were 28 cells (Figure 1A). In the last time slice, we identified 92 cells (Figure 1B). Since the manual tracking started from the last time slice to the first time slice backward, we found 92 marker points of the cell nucleus in each time slice and assigned the ID number. In the cell division event, the cell before the division was defined as the mother and the two cells after division as daughter cells.

Cell division occurs during cell migration. For analysis, it is crucial to identify the correspondence between mother and daughter cells and the time at which cell division occurs. For all the marker points in each time slice, we calculated the Euclidean distance between each pair of marker points. A sudden change in the distance indicates that cell division had taken place (Figure 1C). The time-lapse observation recorded the image slices every 15 min. The distance between the two adjacent slices is defined as the step size. For a representative cell, the step size distribution for all pairs of slices is shown in Figure 1D. The maximum step size was 6 μm. The distances between the four groups of daughter cells are shown in Figure 1E. The graph show a clear “distance jump” in the curve, where the distance before the “jump” is always very short. The “distance jump” indicates the occurrence of cell division. After the “jump” a completely different distance curve appears. The Euclidean distance between the marker points was greater than 10 μm after cell division. The maximum moving step size of 6 μm is below the “jump distance” of 10 μm, which means that the accuracy is acceptable for identifying when cell division occurs. In combination with fluorescence images, the division relationship between cells can be determined. We identified divisions based on the calculation of the distance between the cell marker points and manually labeled cells. The marker points of the mother and two daughter cells were unified prior to cell division.

### 3.2. Dimension Reduction

**Figure 2:**
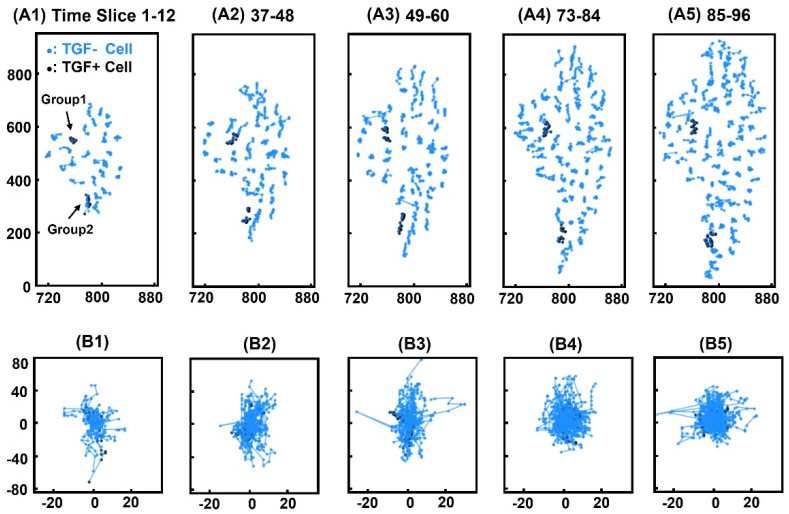
Before and after cell tracks’ positional normalization. (**A1**–**A5**) The positions of TGF (+) (black) and TGF (−) (blue) cells before normalization in different observation windows. (**B1**–**B5**) The positions after normalization, the initial position transferred to (0, 0).

After the cell tracking, the entire migration process was divided into multiple periods. The trajectories of TGF (+) (black) and TGF (−) (blue) cells during different periods are shown in Figure 2(A1–A5). Two groups of TGF (+) cells were observed from the initial position and marked separately. The cell trajectories within each period were normalized. The purpose of normalization is to eliminate the effect of location (Figure 2(B1–B5)), where “normalization” refers to translating all cell trajectories such that their starting points are located at the origin (0, 0).

**Figure 3:**
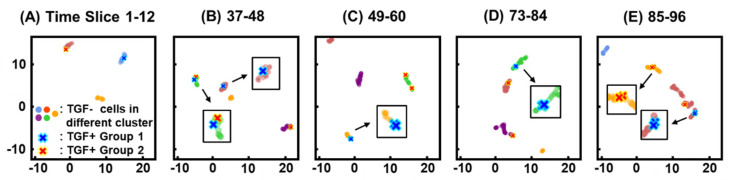
Visualization of cell tracks after dimensionality reduction. (**A**–**E**) 2D visualization of TGF (+) (“X” mark) and TGF (−) cell (colored points) trajectories after dimensionality reduction by UMAP and clustering by k-means. Each mark and point represent a trajectory. Colors represent different clusters. The insets show the magnified images of TGF (+) cells when they overlap (**B**–**E**).

The normalized cell trajectories are used to calculate the distance matrix, which is a series of high-dimensional datasets for which we need to reduce the dimensionality for visualization. There are two reasons for reducing the dimension of the data before performing k-means clustering, in addition to visualization. The first reason is data summarization. Our method aims to find a low-dimensional structure from high- dimensional cell-trajectory data. Reducing the dimensions before performing k-means clustering is beneficial for capturing the low dimensional structures. The second reason is robustness. Dimension reduction can help reduce the noise included in high-dimensional data and improve the robustness of our method. Dimension reduction before performing downstream analysis, such as clustering, is also used in single-cell RNA-sequencing data analysis [34,35]. We chose two as the number of the dimensions to ensure consistency with the visualization. The advantage of UMAP over t-SNE is its ability to reflect both local and global structures while maintaining relative global distances in a low-dimensional space. These capabilities allow us to obtain appropriate clustering results. Figure 3 shows the dimensionality reduction results for several periods with TGF (−) cells (green) and TGF (+) cells (red and blue). We also calculated using the t-SNE for comparison (Figure S1). As a result, UMAP showed a more apparent aggregation of data points in the same cluster. In addition, there were more apparent boundaries between the clusters.

The optimal observation window was selected such that the distance between the clusters was larger in the first and last time periods and the data points within the clusters were closer together (Figure S2). We selected an observation window of 12 time slices. As shown in Figure S3, the parameters for UMAP were chosen, such that the distance between clusters was larger and the data points within the clusters were closer.

### 3.3. Clustering

The clustering method used the k-means algorithm, and for each time period, we used the MSC method to determine the appropriate number of clusters (Figure S4). MSC $\bar{s}\left(k\right)$ is in the range of (−1,1); therefore, the closer $\bar{s}\left(k\right)$ is to 1, the more reasonable the clustering is, at which point *k* is the appropriate number of clusters. The results of the clustering are shown in Figure 3A–E, where the different clusters are labeled with different colors. There were two TGF (+) cells in the first generation that were distant from each other. Multiple daughter cells were observed after cell division. Until the last time slice, five TGF (+) cells were present (refer to Figure S5). According to each division, TGF (+) was referred to as two groups, where Group 1 included TGF (+) Cell ID 4 and 5 and Group 2 included TGF (+) Cell ID 1 to 3.

### 3.4. Similarity of Migration Patterns

In each period in Figure 3A–E, it is clear that TGF (+) cells of the same group were not always in the same cluster at the same time. TGF (+) and TGF (−) cells assigned to the same cluster showed high similarity and maintained the same migration pattern in terms of collective cell migration. TGF (+) cells with more active migratory behavior exert some influence on TGF (−) cells. TGF (+) cells with a leading role showed interactions with TGF (−) cells through signaling. Pathways such as PI3K-Rac signaling are involved in actin remodeling and mediation of collective migration. Through PI3K-dependent integrin adhesion and modulation of Rho-GTPase signaling, cadherin-induced regulation of actomyosin contractility at more distant sites in the cell affects global cellular mechanics [2,36]. The present analysis found that cells around TGF(+) cells showed the same migration pattern, and they might have been under the influence of the leader cell. The behaviors observed here may correspond to reports that the leader cell leads the movement of the cell population such that the surrounding follower cells are in tow. Therefore, our methodology could be used to detect leader cells within a cell population. This maintained them in the same overall migration direction and distance.

In addition to clusters containing TGF (+) cells, some clusters contained only TGF (−) cells, illustrating that their migration pattern is different from that of TGF (+) cells. TGF (+) cells, as leaders of collective migration, affect nearby TGF (−) cells; however, the effect is relatively narrow. TGF (−) cells with fewer or no interactions migrated based on their migratory patterns.

### 3.5. Positional Similarity

**Figure 4:**
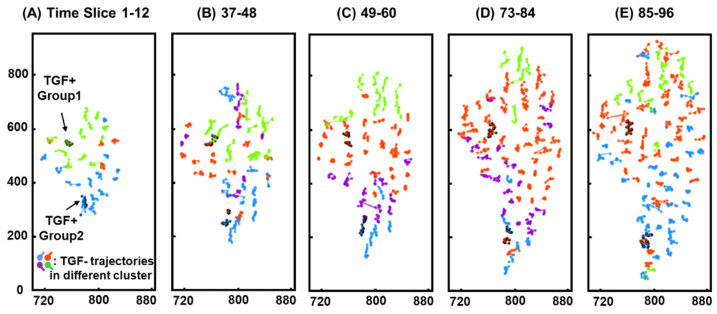
Cell trajectories’ positions combined with clustering results. (**A**−**E**) Two groups of TGF (+) cells are marked black pointed by arrows. Colors represent different clusters.

It is expected that the influence of leader cells on follower cells decreases with distance and is stronger only within a certain range. Within the effective range, the interaction strengthened the connections between them. To verify these results, we superimposed the clustering results onto a cell location map. During the previous normalization process, the location information of the cell trajectories was reset and the similarity of the cell trajectories was determined from the clustering results. However, it is impossible to determine whether cell trajectories with similarities are close to each other in terms of their actual locations. Therefore, the clustering results and actual locations were combined, as shown in Figure 4. Notably, the clustering results in Figure 4 are the same as those in Figure 3; however, the color was adjusted for ease of comparison. The data points for TGF (+) cells in Figure 4 are marked in black, whereas the lines between the points remain colored.

Interestingly, after combining the clustering results with actual locations, most cells from the same cluster showed positional similarities. Our method reproduces positional similarity, even after normalization eliminates the location information. This worked for most cells, except for individual cells, such as the green cluster in Figure 4B, which contained cells belonging to the other clusters.

We used an aligned fiber to mimic the ECM in an in vitro model. This environment closely resembles the environment inside an organism. The migration direction of some cells may be influenced by fiber alignment; however, after normalization, the effect of position was eliminated. Normalization makes the method reliable and the trajectory independent of the ECM. In addition, the calculation of this method treats all cells equally and does not distinguish between leader and follower cells, but the results show cells with similar migration patterns. After superimposing the clustering with the actual locations, we found that in most cases, there were many TGF (−) cells around TGF (+) cells. From these results, we can see that clusters are not related to cells moving in a limited direction; they are related to the similarity of migration. TGF (+) cells were mostly not in the center of each cluster but at the edge of the cluster, which is consistent with the characteristics of the leader cells.

### 3.6. Cell Division

Cell division can occur during migration and may affect the surrounding microenvironment, resulting in altered migration characteristics. The direction and speed of the migration of individual cells in a colony can be altered by cell division. These changes may affect the migration pattern of the colony. Therefore, it is necessary to establish a cell lineage for analysis of cell migration patterns. The cell lineage was drawn to identify the specific time slices where cell division occurred and the correspondence between daughter and mother cells (Figure S5). Cell IDs 1–5 are TGF (+) cells, and the others are TGF (−) cells. The time of cell lineage (Figure S5) was divided into eight periods. The color in each time period (Figure S5) indicates the cluster to which the cell belongs, and the color remains the same as that in Figure 4.

In combination with Figure S5, TGF (+) cells ID 4 of Group 1 were divided into Cell ID 5 at time slice 38, and these cells were always surrounded by TGF (−) cells, although these cells were sometimes not fixed. Cell ID 3 of Group 2 was the first generation of cells, and the following two divisions occurred in time slices 32 and 88. Interestingly, the clusters of TGF (+) Group 2 and the surrounding TGF (−) cells in Figure 4B,D,E were different; the colors do not exactly match those of the surrounding TGF (−) cells. Our explanation for this phenomenon was that the state of the cells changed during cell division, which affected the migration pattern and trajectory of the cells. This led to differences in clustering. The state of cell division lasts for some time; therefore, the effect may occur over multiple periods. In contrast, Group 1 had only one cell division at time slice 38. Therefore, the clustering of Group 1 and the surrounding TGF (−) cells remained consistent for all periods, except for Figure 4B. The clusters of both daughter and parent cells essentially changed (e.g., Figure S5, Cell ID 34, 56, 59). Thus, cell division resets cell-cell interactions and creates a new surrounding environment, which is the key to changing the migration similarity.

### 3.7. Robustness

**Figure 5:**
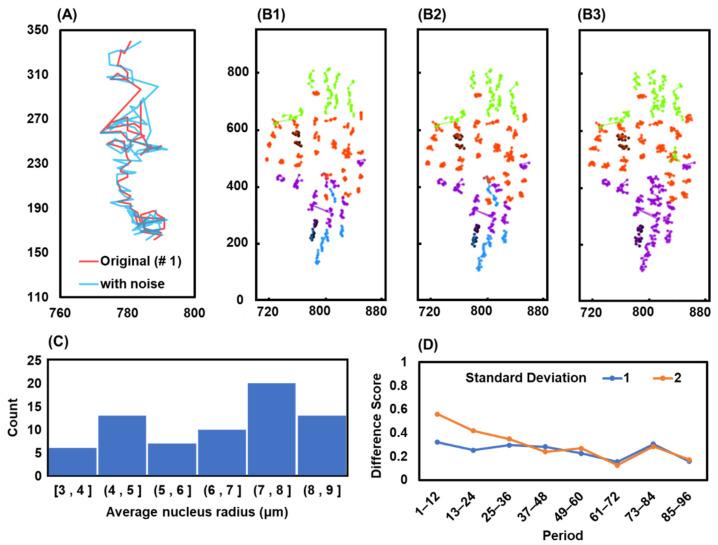
Robustness of the method. (**A**) The original trajectory of Cell ID 1 (red) and the trajectory with Gaussian noise of standard deviation σ = 2 (blue). (**B1**) Cell tracks’ positions combined with clusters’ result under time slice 49–60. (**B2**,**B3**) All trajectories are with noise of standard deviations = 1, 2. (**C**) Histogram of average radius of cell nucleus (μm). (**D**) The difference score of all clustering results under different standard deviations (from the mean of three calculations in each *σ*). The sample sizes for each time period are 92 cells.

Whether this method is effective in identifying cells with similar migration patterns requires further validation. During manual marking, the accuracy of the marked cell nucleus position determines the degree of error. The robustness of the clustering results was verified by adding noise to the cell trajectories. The test data were generated by adding Gaussian noise with a fixed mean and standard deviation, *σ* (Figure 5A). Figure 5(B1) shows the original positions of the cell trajectories for time slices 49–60, and Figure 5(B2,B3) show the clustering results with *σ* = 1 and 2, respectively. A change in *σ* causes the similar trajectories to become dissimilar. The boundaries between clusters became indistinct and *k* increased, rendering the clustering meaningless. The maximum value of *k* for the original clustering results was 5. Therefore, we applied the maximum value of *k* = 5 to noise-containing clustering analysis. The trajectory difference score of the clustering results with noise compared to those without noise is shown in Figure 5D. Notably, the cluster color order (cluster index) changed after each adjustment of the *σ* value. Therefore, we recorded the ID of each trajectory in each cluster and then adjusted the color order as a whole to ensure reasonableness (Figure 5(B1–B3)).

As shown in Figure 5(B1–B3), the distribution of cluster positions did not differ significantly and only the clusters of individual cells changed. The observation error is mainly due to the accuracy of manual labeling. The radius of the nucleus was below 9 μm (Figure 5C). Considering the accuracy the manual labeling of the center of the cell nucleus, *σ* = 2 was sufficiently large for our test. The clustering results with noise and without noise were comparable in consistency. This difference may be related to noise altering the original migration trajectory. This is because the change in trajectory affects the migration pattern, including speed and direction. The internal connections between cells that originally belonged to the same cluster are broken. When *σ* is greater, noise causes severe distortion of the trajectory. Nevertheless, there were no significant difference in the position of each cluster. Therefore, the method is robust to observation errors that may be contained in the cell trajectory data.

## 4. Conclusions

Interactions between cells are essential for coordinated and directed collective movements. Although, collective cell migration has been extensively studied, this study focused on exploring the similarity of migration patterns based on migration trajectories. Using a combination of dimensionality reduction and clustering techniques, cells with similar migration patterns were observed to exhibit positional similarities. Cell division is an important factor that affects the migration patterns. TGF (+) and TGF (−) cells belonging to the same cluster showed similar migration patterns and, within a certain range, migration was consistent. Validation by adding noise illustrated the robustness of the proposed method. This provides a new perspective for a deeper understanding of collective cell migration. The method applies to collective cell migration on aligned fibers and models such as wound healing and migration on other substrates. It can also be extended to the cell migration model in a 3D hydrogel matrix if cells can be tracked, indicating its application in tissue engineering and organ development research.

## Data Availability

The code to generate the data is available on the GitHub repository: https://github.com/CollectiveCellMigration/CellTrajectoryClustering (accessed on 15 September 2022).

## Supplementary Materials

The following supporting information can be downloaded at: https://www.mdpi.com/article/10.3390/cancers14194587/s1, Figure S1. The dimensionality reduction of cell trajectories by t-SNE under different parameters and observation windows. Figure S2. Cell tracks after dimensionality reduction when select different observation windows. The first and last periods showed that window 12 has clear boundaries of clusters and TGF (+) cells in the same group tend to the same cluster. Figure S3. The dimensionality reduction of cell trajectories under different UMAP parameters (observation window = 12, time slice 85–96). Figure S4. WCSS (within-cluster sum of squares of distance) (blue) and mean silhouette coefficient (MSC) (orange) of all samples with the number of k-means clusters of UMAP dimensionality reduction results under different observation periods. The K_p_ value shown by the red arrow is the optimal number of clusters. The initial number of TGF (+) and TGF (−) cells at each period is included in each figure. There were 5 TGF (+) cells and 87 TGF (−) at the last slice (slice 96). Figure S5. Cell lineage tree. Daughter cells are divided from mother cells at different time slice (red font). Colors represent the same clusters as in Figure 5. Cell IDs 1–5 are TGF (+) cells (Group 1: ID 4–5, Group 2: ID 1–2), Cell ID 6–93 are TGF (−) cells. Movie S1. NMuMG cells migration on the PS fibers. TGF (+) cells (red labeled) and TGF (−) cells (green labeled).
