# Supplementary material for: Time-Series Clustering of Single-Cell Trajectories in Collective Cell Migration
Source: Cancers (Basel). 2022 Sep 22;14(19):4587. doi: 10.3390/cancers14194587 (PMC9559181; doi:10.3390/cancers14194587)
Supplement: Supplementary file 1 [file cancers-14-04587-s001.zip › Support_Xin_Rev.pdf]

SUPPORTING INFORMATION

Time-series Clustering of Single-Cell Trajectories in Collective Cell Migration

Zhuohan Xin [1†], *Masashi K. Kajita* [2,3†], *Keiko Deguchi* [4], *Shin-ichiro Suye* [1,3,4] *and Satoshi Fujita* [1,3,4*]

[1] Department of Advanced Interdisciplinary Science and Technology, University of Fukui, Fukui, 910-8507, Japan

[2] Department of Applied Chemistry and Biotechnology, University of Fukui, Fukui, 910-8507, Japan

[3] Research and Education Program for Life Science, University of Fukui, Fukui, 910-8507, Japan

[4] Department of Frontier Fiber Technology and Science, University of Fukui, Fukui, 910-8507, Japan

***Corresponding author**

Satoshi Fujita

Department of Frontier Fiber Technology and Science, University of Fukui, 3-9-1, Bunkyo, Fukui 910-8507, Japan

Tel: +81-776-27-9969, Fax: +81-776-27-9969, E-mail: fujitas@u-fukui.ac.jp

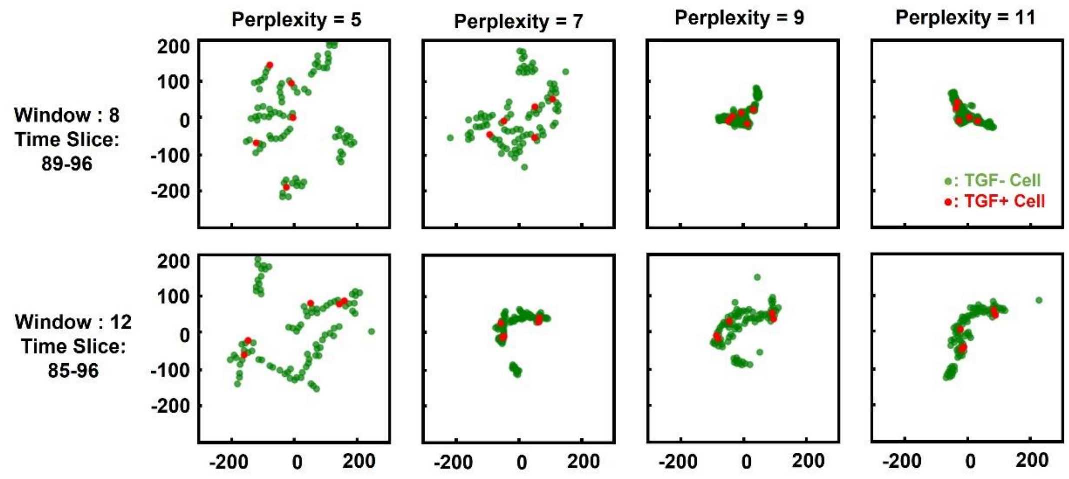

Figure S1. The dimensionality reduction of cell trajectories by t-SNE under different parameters and observation windows.

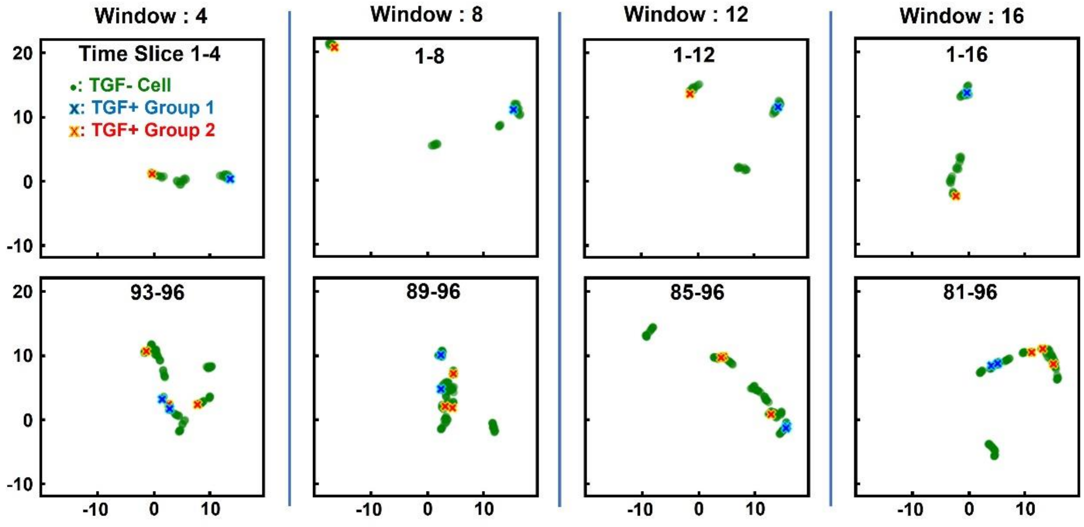

Figure S2. Cell tracks after dimensionality reduction when select different observation windows. The first and last periods showed that window 12 has clear boundaries of clusters and TGF (+) cells in the same group tend to in the same cluster.

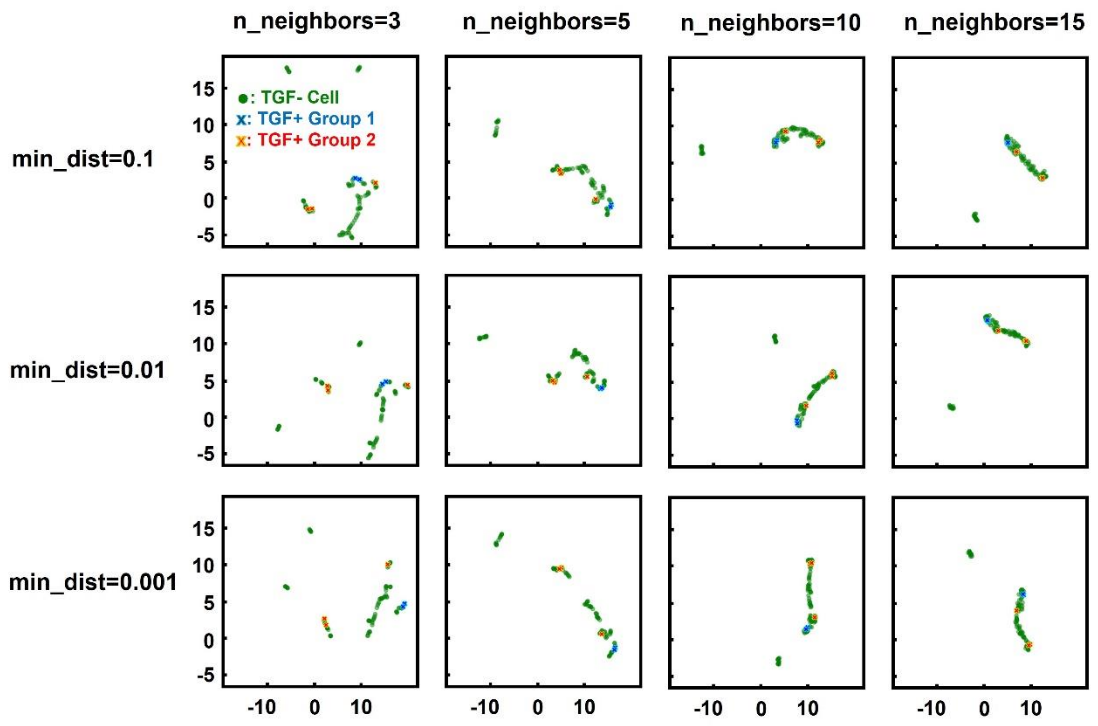

Figure S3. The dimensionality reduction of cell trajectories under different UMAP parameters (observation window = 12, time slice 85-96).

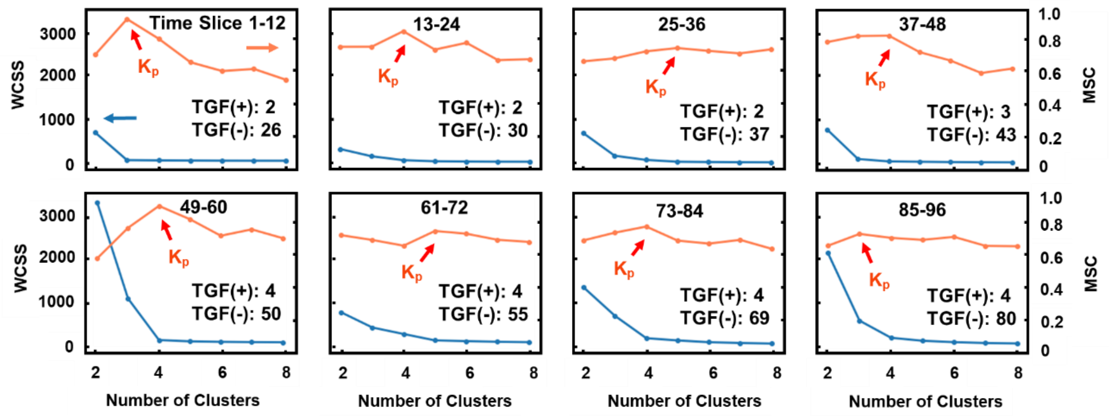

Figure S4. WCSS (within-cluster sum of squares of distance) (blue) and Mean silhouette coefficient (MSC) (orange) of all samples with the number of K-Means clusters of UMAP dimensionality reduction results under different observation periods. The K_p value shown by the red arrow is the optimal number of clusters. The initial number of TGF (+) and TGF (-) cells at each period is included in each figure. There were 5 TGF (+) cells and 87 TGF (-) at the last slice (slice 96).

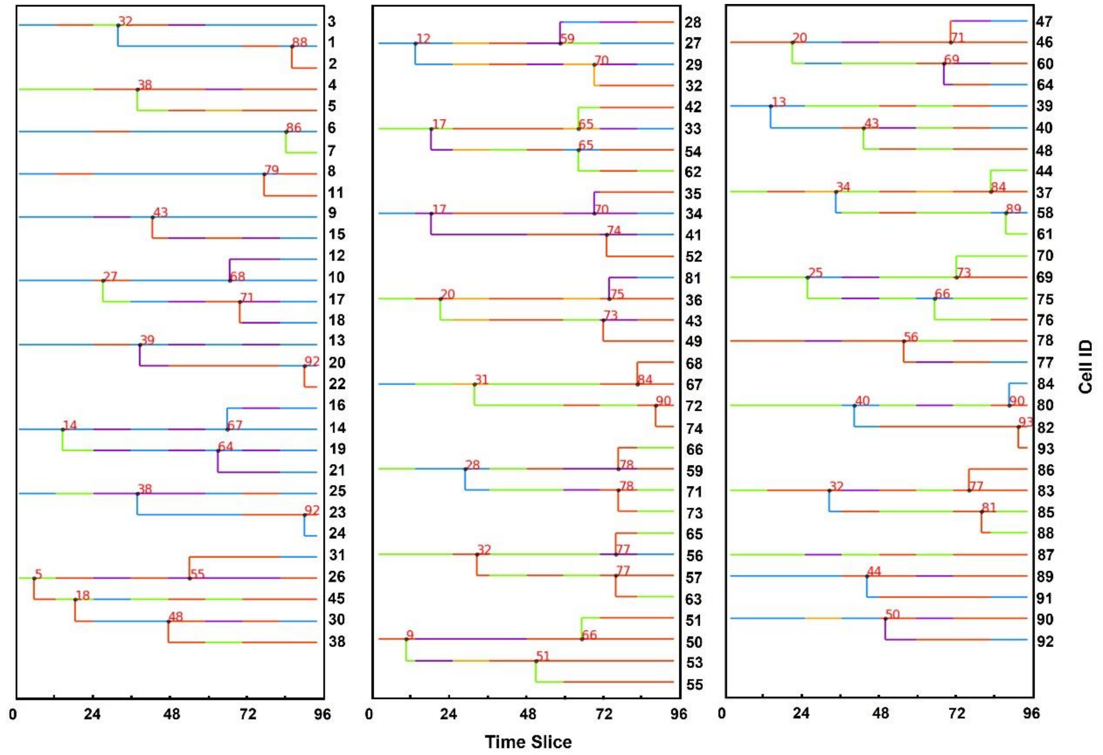

Figure S5. Cell lineage tree. Daughter cells are divided from mother cells at different time slice (red font). Colors represent the same clusters as in Fig.5. Cell ID 1-5 are TGF (+) cells (Group1: ID 4-5, Group2: ID 1-2), Cell ID 6-93 are TGF (-) cells.

Movie S1. NMuMG cells migration on the PS fibers. TGF (+) cells (red labeled) and TGF (-) cells (green labeled).
